# High Rate of Gastrointestinal Bleeding in Patients with Secondary Sclerosing Cholangitis in Critically Ill Patients (SC-CIP)

**DOI:** 10.3390/jcm10091925

**Published:** 2021-04-29

**Authors:** Andreas Blesl, Martin Eibisberger, Michael Schörghuber, Christoph Klivinyi, Vanessa Stadlbauer

**Affiliations:** 1 Department of Internal Medicine, Division for Gastroenterology and Hepatology, Medical University of Graz, 8036 Graz, Austria; vanessa.stadlbauer@medunigraz.at; 2 Department of Radiology, Medical University of Graz, 8036 Graz, Austria; martin.eibisberger@medunigraz.at; 3 Department of Anesthesiology and Intensive Care Medicine, Medical University of Graz, 8036 Graz, Austria; michael.schoerghuber@medunigraz.at (M.S.); christoph.klivinyi@medunigraz.at (C.K.)

**Keywords:** secondary sclerosing cholangitis in critically ill patients, SC-CIP, gastrointestinal bleeding, critically ill, cholestatic liver disease

## Abstract

Secondary sclerosing cholangitis in critically ill patients (SC-CIP) is a rare cholestatic liver disease triggered by long-term intensive care treatment. The aim of this study was to evaluate the frequency and characteristics of gastrointestinal bleeding in SC-CIP. Patients with diagnosed SC-CIP were retrospectively identified and compared to a control group of patients with cardiac surgery and intensive care treatment but without the development of SC-CIP. Fifty-three patients with SC-CIP and 19 controls were included in the study. The frequency of gastrointestinal bleeding was 30% in SC-CIP (16 patients) and 5% in the control group (1 patient) (*p* = 0.03). Bleeding occured in the mean 13 months after admission to an intensive care unit in SC-CIP, three patients (19%) suffered bleeding during intensive care treatment. Three SC-CIP patients (19%) had cirrhosis at the time of bleeding, five (31%) had splenomegaly, and four (25%) received oral anticoagulation. In SC-CIP, 13 bleedings were identified in the upper gastrointestinal tract, two in the lower, and one remained unknown. The most common reasons for bleeding were gastroduodenal ulcers. In total, 80% of patients needed blood units, and one death due to bleeding occurred in SC-CIP. In conclusion, gastrointestinal bleeding is a frequent complication in patients with SC-CIP. Whether the liver disease itself or cofactors cause the susceptibility for bleeding remains unclear.

## 1. Introduction

Long-term intensive care treatment with invasive ventilation and hemodynamic support may lead to secondary sclerosing cholangitis in critically ill patients (SC-CIP), a relatively rare chronic cholestatic liver disease. It occurs in patients without prior known liver pathology and predominantly in patients after polytrauma or cardiovascular surgery and with pre-existing internal pathologies [1,2,3]. Recently, a case after a severe SARS-CoV-2 infection with multiorgan failure was reported [4].

The pathogenesis of SC-CIP remains unclear. Ischemic cholangiopathy is discussed as the main triggering factor [1,5]. Other than hepatocytes, which receive perfusion from the portal vein and the hepatic arteries, blood flow for the biliary epithelium is supplied solely by branches of the hepatic arteries. This makes the biliary tree more vulnerable to ischemic injury [6,7]. Further cofactors in the evolution of SC-CIP include recurrent biliary infections and the formation of bile cast. Candida and bacteria are found in the bile of nearly every patient with SC-CIP [1]. The development of the disease seems to be favored by NOD2 gene variants [8]. Additionally, our group recently described alterations of the gut microbiome, intestinal permeability, and serum bile acid profiles in SC-CIP, which may not be caused by the liver disease alone, but seem to persist also from long-term intensive care treatment [9].

SC-CIP leads to destruction of the intra- and extrahepatic biliary tree with the development of biliary strictures that may lead to liver fibrosis and consequently to cirrhosis of the biliary type. Prognosis is generally poor, and progression to cirrhosis within months can occur. Liver transplantation is the only curative treatment option [1].

Gastrointestinal (GI) bleeding occurs in 1.2 to 14% of patients in an intensive care unit [10,11,12,13]. The main reasons for bleeding are ulcers and erosions in the upper GI tract [11]. Furthermore, upper GI bleeding is a frequent complication of liver cirrhosis [14]. The primary bleeding causes comprise esophageal and gastric varices due to portal hypertension [15]. Although asymptomatic peptic ulcers are frequently found in cirrhotic patients, bleeding from ulcers is less common than from varices, even though it can be favored by coagulopathy as a part of hepatic dysfunction [16,17,18,19]. Portal hypertensive gastropathy (PHG) is the third most frequent bleeding cause in cirrhosis. Rare causes include Mallory–Weiss syndrome, Dieulafoy’s lesions, erosive gastritis, esophagitis, and gastric vascular ectasia [20,21]. Dieulafoy’s lesions are more common in patients with advanced liver disease [22,23]. In lower GI bleeding of cirrhotic patients, hemorrhoids and portal hypertensive enteropathy or colopathy were found to be the main reasons for bleeding [24]. The occurrence of GI bleeding in noncirrhotic patients suffering from chronic liver diseases has not yet been studied in detail. Furthermore, there is no data available about the frequency of GI bleeding in patients with SC-CIP.

Therefore, we aimed to conduct the present retrospective study to evaluate the frequency and the causes of GI bleeding in patients with SC-CIP. We included a large cohort of SC-CIP patients and a control group of patients with history of treatment in an intensive care unit (ICU) but without development of SC-CIP.

## 2. Materials and Methods

### 2.1. Study Population

Patients with diagnosis of SC-CIP were identified from the medical records of the Medical University of Graz starting in May 2020 and were followed retrospectively from time of the initial incident leading to SC-CIP to death or to November 2020, if still alive. The diagnosis of SC-CIP was established by clinical suspicion and by typical cholangiographic findings, including irregular intrahepatic bile ducts with strictures, prestenotic dilations, bile duct rarefication, and biliary casts on magnetic resonance cholangiopancreatography (MRCP), on endoscopic retrograde cholangiopancreatography (ERCP), or with liver biopsy. Exclusion criteria was cholestasis of other origins, such as choledocholithiasis, primary sclerosing cholangitis, primary biliary cholangitis, IgG4-associated cholangitis, or toxic cholestasis. All included patients had negative history of liver diseases before ICU admission. Viral hepatitis, hereditary and metabolic liver diseases, and autoimmune liver disease were excluded serologically. To exclude autoimmune hepatitis and distinct chronic cholestatic liver diseases, the following parameters were assessed: antinuclear antibodies (ANA)—measured with indirect immunofluorescence, anti-mitochondrial antibodies (AMA), anti-smooth muscle antibodies (SMA), anti-liver-kidney microsomal antibodies (anti-LKM), anti-neutrophil cytoplasmatic antibodies (ANCA), and immunoglobulins G, A, and M.

A control group of patients with the need for prolonged intensive care treatment after cardiac surgery who were considered at risk for SC-CIP but did not develop the disease were initially recruited prospectively and were monitored retrospectively for the present study for the occurrence of GI bleeding.

### 2.2. Definitions

Bleeding was noted when any signs of GI hemorrhage occurred. This included distinct hematochezia, melena, or hematemesis as well as active or suspended bleeding upon endoscopy, obvious bleeding on angiography, and occult bleeding if assessed.

### 2.3. Primary Endpoint

The primary endpoint was the occurrence of GI bleeding within the observation period.

### 2.4. Secondary Endpoints

Secondary endpoints were the assessment of bleeding characteristics, the outcome of patients, and the evaluation of the vascular anatomy of SC-CIP patients to check for alterations increasing the bleeding risk.

### 2.5. Data Collection

Patients with SC-CIP were identified through a keyword search in the medical documentation system of the Medical University of Graz. SC-CIP, secondary sclerosing cholangitis, and sclerosing cholangitis were used as keywords. Patients with primary sclerosing cholangitis were excluded, and all identified patients with a definite diagnosis of SC-CIP were included in the analysis. The medical records were then checked for the occurrence of GI bleeding, and bleeding characteristics and outcomes were documented. All this work was solely done by the first author. Patients of the control group were identified through an ongoing prospective study (currently on hold due to the COVID-19 pandemic) intended to assess the prevalence of SC-CIP in cardiac surgery patients. Patients after cardiac surgery who needed mechanical ventilation +/- extracorporeal membrane oxygenation for five days or more were included. Those who did not develop SC-CIP were selected as control group for the present study. The gathering of additional information and evaluation of bleeding events was done by the first author.

### 2.6. Radiological Evaluation

The evaluation of vascular anatomy and the presence of vascular stenosis in SC-CIP patients was done by a radiologist using cross-sectional imaging, depending on availability (contrast-enhanced and -unenhanced computer tomography, contrast-enhanced and -unenhanced magnetic resonance tomography, computer tomography angiography, and magnetic resonance angiography). The chosen images for interpretation were the closest ones available to the initial incident leading to ICU treatment. All images were taken due to reasons associated with necessary medical care, none of them for the purposes of the study.

### 2.7. Statistical Analysis

Statistical analysis was done using SPSS version 25. A Kolmogorov–Smirnov test was used to check for normal distribution. Descriptive statistics were used for the description of variables; the Chi-squared test, Student’s T-test, and Mann–Whitney U test were used to check for significance. All tests were performed on a 5% significance level.

### 2.8. Ethical Considerations

Data were obtained from two studies, both approved by the research ethics committees at the Medical University of Graz (protocols 26-569 ex 13/14 and 30-342 ex 17/18) and registered at clinicaltrials.gov (NCT02545309 and NCT03566797). The studies were performed in accordance with the ethical standards laid down in the Declaration of Helsinki and its amendments.

## 3. Results

### 3.1. Patient Characteristics

Fifty-three patients with SC-CIP and 19 controls were included in the analysis. Baseline characteristics are shown in Table 1 and Table 2. The mean age at the study inclusion was 57 ± 14 (mean ± standard deviation) years in SC-CIP and 73 ± 10 years in the control group (*p* < 0.001). One quarter of the patients in both groups were female (SC-CIP: 14 (26%), controls: 4 (21%)). The length of ICU stay was comparable between the two groups (*p* = 0.1). The reasons for intensive care treatment in SC-CIP included internal medicine emergencies, such as myocardial infarction and acute respiratory distress syndrome, as well as burns and polytrauma. The diagnosis of SC-CIP was made 12 ± 22 months after admission to the ICU. Patients in the control group were hospitalized due to acute myocardial infarction, aortic dissection, or valvular heart disease with the need for cardiac surgery. In SC-CIP, 9 patients (17%) needed liver transplantation within the observation period, and 16 patients (30%) died, mostly due to complications related to the initial incident leading to ICU treatment. In the control group, six deaths (32%) occurred. Mean follow-up was nearly 5 years in SC-CIP and 1.5 years in the control group. In total, 3053 patient months (254 years) in SC-CIP and 296 patient months (25 years) in the control group were overviewed.

### 3.2. Frequency of GI Bleeding

GI bleeding occurred in 16 SC-CIP patients (30%) and 1 patient (5%) of the control group (*p* = 0.03). In SC-CIP, bleeding occurred in the mean 13 ± 19 months after ICU admission, 10 patients suffered bleeding within the first year after ICU admission (data not available for two patients) and three bleeding episodes occurred during the ICU stay (Table 3). Only three patients (19%) had cirrhosis at the time of bleeding, five (31%) had splenomegaly, and four (25%) received oral anticoagulation (indications: atrial fibrillation, mechanical mitral valve, thrombus of the left ventricle, bypass surgery). Platelets were significantly (*p* = 0.01), GGT (*p* = 0.1) and AP (*p* = 0.4), numerically lower at bleeding than at the diagnosis of SC-CIP, while bilirubin (*p* = 0.1) was numerically higher at bleeding. Most patients presented with normal prothrombin time during bleeding. Bleedings were severe in SC-CIP, with hemoglobin levels of 8.0 ± 1.7 g/dL at the time of diagnosis of GI bleeding.

### 3.3. Characteristics of GI Bleeding in SC-CIP

**Figure 1:**
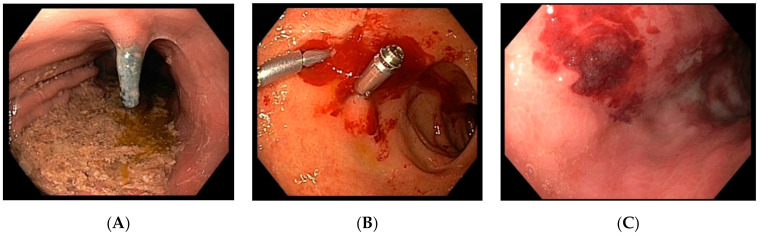
Bleeding sources in three patients included in the study. (**A**) Patient 7. Second look endoscopy after suspension of bleeding. Endoscopy showed a hemoclip in the body of the stomach at the small curvature. The bleeding originated from a visible vessel from an ulcer at an atypical location. (**B**) Patient 16. The endoscopy revealed active bleeding from an ulcer located in the duodenal bulb. The bleeding was rated as Forrest 1b. Hemostasis was finally achieved with hemoclips. (**C**) Patient 9. Endoscopy revealed several ulcers in the stomach with one showing bleeding signs (coagulum) rated as Forrest 2b. As no active bleeding was obvious, the endoscopist did not intervene, and conservative therapy with proton pump inhibitors was initiated.

In SC-CIP, bleeding in only two patients originated from the colon (ulcers in the rectum of unknown origin in one patient, atypical visible vessel without ulceration in the ascending colon in the other patient). In 13 patients, bleeding locations were identified in the upper GI tract. Most bleedings originated from ulcers located in all parts of the upper GI tract (see bleeding characteristics, Table 4). Notably, ulcers in the stomach occurred at atypical locations for peptic ulcers (see examples, Figure 1), and visible vessels also occurred without ulceration (Dieulafoy’s lesions). In one patient, the bleeding source could not be detected. Eight patients were evaluated with gastric biopsies for the presence of helicobacter pylori; all were negative. As hemostatic methods, the application of submucosal diluted adrenalin, hemoclips, hemostatic gel, argon plasma coagulation (APC), and hemostatic powder were used. One patient needed coiling via angiography due to unsuccessful endoscopic intervention. Supportive medical treatment with proton pump inhibitors (PPI) was used in all patients. Dosing was inhomogeneous, ranging from 40 mg once daily to 80 mg three times per day or continuous application. In total, 80% of patients needed blood units, six needing more than five. One patient died as a consequence of GI bleeding; he suffered from SC-CIP-induced liver cirrhosis.

### 3.4. Characteristics of GI Bleeding in the Control Group

The only bleeding episode recorded in the control group was a positive test for occult blood in the stool in one patient without additional bleeding signs. Suspected bleeding stopped spontaneously, and endoscopic evaluation was not performed.

### 3.5. Radiological Evaluation

Altered vascular anatomy or suspected vascular stenosis occurred with comparable frequency in SC-CIP patients with and without GI bleeding (SC-CIP with bleeding: altered anatomy: *n* = 5 (31%), stenosis: *n* = 1 (6%); SC-CIP without bleeding: altered anatomy: *n* = 8 (24%), stenosis: *n* = 5 (15%) (data of three patients missing)) (Table 5). The variants most frequently observed were modified origins of hepatic arteries. Furthermore, stenosis of the coeliac trunk, the common hepatic artery, and the superior mesenteric artery were observed. Stenosis occurred due to arteriosclerosis, and in one case, innate compression of the coeliac trunk by the diaphragm was suspected. A specific risk pattern for GI bleeding could not be observed.

## 4. Discussion

SC-CIP occurs after long-term intensive care treatment in patients without prior liver pathology. Due to the relatively low number of affected patients, data on the pathogenesis and the very heterogeneous course of the disease remain widely enigmatic [1]. In the present retrospective study, we focused on the frequency of GI bleeding in SC-CIP. Our study suggests for the first time that GI bleeding arises in nearly one out of three SC-CIP patients and that bleeding is more common in SC-CIP than in patients with history of long-term intensive care treatment without the emergence of SC-CIP.

GI bleeding, often caused by gastroduodenal ulcers, occurs frequently in critically ill patients [11,12,25]. For this reason, stress ulcer prophylaxis is widely used, although evidence concerning its benefit on mortality remains conflicting [26,27,28]. Coagulopathy, respiratory failure, acute renal failure, liver diseases, and male sex were identified as risk factors for bleeding [11,25]. Long-term ICU treatment as the main driver for the high rate of bleeding in our cohort is conceivable, but only three patients suffered bleeding during ICU treatment and the total rate of 30% in SC-CIP is twice as high as previously reported in critically ill patients in a study from the pre-PPI era [12]. Because GI bleedings were counted over a longer period, comprising not only the ICU stay and the following inpatient treatment in our cohort, data are not completely comparable. The interval of ICU admission to bleeding was more than a year in the mean of SC-CIP, and bleeding also occurred long after ICU treatment. In the control group, only one minor bleeding occurred. Whether the disease itself favors the occurrence of GI bleeding or whether our cohort presents a negative selection of particularly ill patients cannot be answered definitively due to the retrospective design of the study. However, since patients in the control group also suffered from severe illnesses, such as aortic dissection, with long-term ICU treatment after emergency surgery, but experienced significantly fewer bleeding episodes, this may favor the hypothesis that SC-CIP is a risk factor for GI bleeding.

Patients with liver cirrhosis are at risk of GI bleeding; the risk in patients with liver fibrosis is yet unknown [14]. The main reason is portal hypertension with a portal venous pressure over 10 mmHg leading to esophageal and gastric varices and portal hypertensive gastropathy [15,29]. Remarkably, in our SC-CIP cohort, cirrhosis and signs of portal hypertension (splenomegaly, thrombopenia) were present in under 30% of patients at time of bleeding. Varices as bleeding source were not reported. Consequently, an increased bleeding risk in SC-CIP may not be caused solely by portal hypertension.

Upper peptic GI tract ulcers account for 30 to 60% of nonvariceal upper GI bleeding and for 20 to 30% of GI bleedings in liver cirrhosis [14,30,31]. Higher mortality rates of peptic ulcer bleeding have been reported in patients with cirrhotic and noncirrhotic liver diseases [17,22,32]. Cirrhotic patients are at short-term risk for recurrent in-hospital bleeding, and increased long-term re-bleeding risk is discussed [22,30,33]. In SC-CIP, ulcers were identified as the main bleeding source (over 55%). Risk factors for GI bleeding from peptic ulcers include the intake of nonsteroidal anti-inflammatory drugs (NSAIDs), an age of more than 60 years, and the co-administration of aspirin, antiplatelet agents, or steroids [34,35,36,37]. Taking these factors into account, one would expect a higher rate of bleeding in our control group due to increased age and the common use of antiplatelet therapy and anticoagulants in cardiac surgery patients. The use of NSAIDs and steroids during the ICU stay could not be sufficiently assessed in this study, but normally great caution is exercised in patients with severe liver diseases, and NSAIDs are rarely used.

Ischemic damage of the biliary system is discussed as the main driving factor for the development of SC-CIP and was observed in liver biopsies [1,2,6,7,38]. Because of its singular supply from the hepatic artery, the biliary tree is vulnerable to hypotension [39,40,41]. In one patient described in literature, sclerosing arteriopathy with intima thickening became evident in the histology after liver explantation [7]. The vascular supply of the liver is heterogeneous, and only 80% of people have normal anatomy of the hepatic artery [42,43]. Whether certain anatomic variants are more prone to the development of SC-CIP and whether these anatomic variations also alter the vascular supply of the stomach, duodenum, and predestine for GI bleeding remains unknown. In the present study, we aimed to assess vascular anatomy and found that only about 60% of patients with SC-CIP had normal vascular status, but specific alterations could not be associated with an increased bleeding risk. Portal hypertension with collateral circulation was not associated with bleeding in our cohort. As a limitation, available cross-sectional images were not taken for examination of vascular pathology, hence assessment of smaller arteries, vascular malformations, and exact stenosis quantification was not possible.

The mortality of GI bleeding was lower in SC-CIP (6%) than reported for GI bleeding in liver cirrhosis (8–24%) and comparable to noncirrhotic cohorts (2–14%) [14,31,44,45,46,47,48], which is in accordance with the low rate of cirrhosis in our cohort.

Our study has the following limitations: as a retrospective study, data quality may vary and occurrence of bleeding may be underreported since bleeding episodes may not be documented in the medical information system used for this study when happening in hospitals not connected to this system. Furthermore, the number of patients in the control group was rather small, and the control group differs in some baseline characteristics from the SC-CIP group. Additionally, the time of observation was shorter in the control group than in SC-CIP (1.5 years vs. 5 years), but most bleedings in the SC-CIP cohort happened within the duration of the observation period of the control group.

The strengths of our study comprise the high number of patients with SC-CIP included in the analysis, the careful data assessment, and the long observation period of SC-CIP.

## 5. Conclusions

In conclusion, data generated with this study suggests high frequency of GI bleeding in SC-CIP with partially atypical bleeding locations and sources. The association with portal hypertension or long-term ICU treatment alone does not seem to be a sufficient explanation for this finding. Abnormal vascular supply of the upper GI tract in SC-CIP patients leading to an increased risk for GI bleeding is the hypothesis established by our study group, but it could not be verified in our cohort. Additional larger, prospective, multicenter studies are needed to validate the increased bleeding risk in SC-CIP and to identify the pathophysiological background.

## Figures and Tables

**Table 1:** Baseline characteristics of patients with SC-CIP included in the analysis.

	SC-CIP (*n* = 53)
Age at study inclusion (years); mean (± SD)	57 (± 14)
Female; *n* (%)	14 (26)
Deaths; *n* (%)	16 (30)
Age at death (years); mean (± SD)	64 (± 11)
Length of ICU stay (days); mean (± SD)	40 (± 22)
Platelets at diagnosis (10^9^/L); mean (± SD)	280,800 (± 132,000)
Bilirubin at diagnosis (mg/dL); mean (± SD)	5.8 (± 7.4)
Alkaline phosphatase at diagnosis (U/L); mean (± SD)	701 (± 499)
GGT at diagnosis (U/L); mean (± SD)	1022 (± 726)
Prothrombin time at diagnosis (%); mean (± SD)	84 (± 27)
Albumin at diagnosis (g/dL); mean (± SD)	3.6 (± 0.8)
Creatinine at diagnosis (mg/dL); mean (± SD)	1.4 (± 1.1)
Liver transplantation; *n* (%)	9 (17)
GI bleeding; *n* (%)	16 (30)
Follow up (months); mean (± SD)	58 (± 47)

SC-CIP = secondary sclerosing cholangitis in critically ill patients; SD = standard deviation; ICU = intensive care unit; GI = gastrointestinal; GGT = gamma-glutamyltransferase.

**Table 2:** Baseline characteristics of patients included in the control group. All patients were treated in the intensive care unit because of cardiac surgery.

	Controls (*n* = 19)
Age at study inclusion (years); mean (± SD)	73 (± 10)
Female; *n* (%)	4 (21)
Deaths; *n* (%)	6 (32)
Age at death (years); mean (± SD)	60 (± 16)
Length of ICU stay (days); mean (± SD)	32 (± 27)
GI bleedings; *n* (%)	1 (5)
Follow up (months); mean (± SD)	16 (± 11)

SD = standard deviation; ICU = intensive care unit; GI = gastrointestinal.

**Table 3:** Characteristics of patients with SC-CIP and GI bleeding.

	SC-CIP (*n* = 16)
Interval ICU-GI bleeding (months); mean (± SD)	13 (± 19)
Interval SC-CIP diagnosis-GI bleeding (months); mean (± SD)	11 (± 23)
Bleeding during ICU stay; *n* (%)	3 (19)
Cirrhosis at GI bleeding; *n* (%)	3 (19)
Splenomegaly at GI bleeding; *n* (%)	5 (31)
Signs of portal hypertension upon endoscopy; *n* (%)	2 (13)
Anticoagulation at GI bleeding; *n* (%)	4 (25)
Hemoglobin at GI bleeding (g/dL); mean (± SD)	8.0 (± 1.7)
Platelets at GI bleeding (10^9^/L); mean (± SD)	173,000 (± 116,000)
Bilirubin at GI bleeding (mg/dL); mean (± SD)	8.0 (± 7.0)
Alkaline phosphatase at GI bleeding (U/L); mean (± SD)	614 (± 559)
GGT at GI bleeding (U/L); mean (± SD)	671 (± 494)
Prothrombin time at GI bleeding (%); mean (± SD)	70 (± 31)
Albumin at GI bleeding (g/dL); mean (± SD)	2.6 (± 0.6)
Creatinine at GI bleeding (mg/dL); mean (± SD)	1.4 (± 0.7)

SC-CIP = secondary sclerosing cholangitis in critically ill patients; ICU = intensive care unit; GI = gastrointestinal; SD = standard deviation; GGT = gamma-glutamyltransferase.

**Table 4:** Characteristics of GI bleeding in SC-CIP patients. All patients with GI bleeding were numbered from 1 to 16. Patient 13 died because of GI bleeding. In all other 15 patients, GI bleeding was not lethal. ICU—bleeding is the time from admission to ICU to the onset of gastrointestinal bleeding in months. Hemoglobin levels were documented at time of diagnosis of gastrointestinal bleeding.

Patient	ICU—Bleeding	Hemoglobin (g/dL)	Location of Bleeding	Bleeding Source	Therapy	Blood Units
1	69	7.3	Duodenum	Ulcer	PPI	3
2	1	8.6	Esophagus, Stomach	Hemorrhagic mucosa	PPI	No
3	31	8.6	Stomach	Ulcer	PPI	1
4	18	4.9	Unkown	Unknown	PPI	> 5
5	2	7.5	Rectum	Ulcers	PPI, HG	Unknown
6	2	11.0	Duodenum	Angiodysplasia, trauma of CBD stent	PPI, APC	No
7	2	5.3	Stomach	Ulcer, Visible vessel	PPI, A, HC	2
8	1	11.2	Esophagus, Stomach	Ulcers	PPI	> 5
9	unknown	unknown	Esophagus, Stomach	Ulcers	PPI	No
10	33	7.3	Duodenum	Bleeding from papilla after EPT	PPI, A	3
11	8	8.8	Esophagus	Coagulum ora serrata, no visible lession	PPI, A	3
12	2	8.8	Duodenum	Ulcers	PPI, Coiling (IR)	> 5
13	unknown	7.6	Stomach	Ulcer	PPI, HC	> 5
14	9	8.1	Colon ascendens	Visible vessel without ulceration	PPI, HC	> 5
15	0	8.1	Duodenum	Visible vessel without ulceration	PPI, A, HC, HP	> 5
16	9	6.6	Duodenum	Ulcer	PPI, HC	4

GI = gastrointestinal; SC-CIP = secondary sclerosing cholangitis in critically ill patients; ICU = intensive care unit; CBD = common bile duct; PPI = proton pump inhibitor; A = adrenalin; HC = hemoclip; HG = hemostatic gel; APC = argon plasma coagulation; HP = hemostatic powder; IR = interventional radiology.

**Table 5:** Description of alterations of the vascular anatomy of SC-CIP patients indicating standard variants and vascular stenosis. The SC-CIP cohort is divided by the occurrence of GI bleeding.

	SC-CIP without	SC-CIP with
	GI Bleeding (*n* = 37)	GI Bleeding (*n* = 16)
Imaging not available	*n* = 3	*n* = 0
Normal vascular anatomy/no stenosis	*n* = 21	*n* = 10
RHA arising from superior mesenteric artery	*n* = 3	*n* = 2
LHA arising from left gastric artery	*n* = 2	*n* = 1
LHA and RHA arising from coeliac trunk	*n* = 1	*n* = 0
LHA and RHA arising from coeliac trunk, gastroduodenal artery arising from LHA	*n* = 1	*n* = 1
Common hepatic artery arising from the aorta	*n* = 0	*n* = 1
Accessory renal artery	*n* = 1	*n* = 0
Stenosis of the coeliac trunk	*n* = 3	*n* = 1
Stenosis of the common hepatic artery	*n* = 1	*n* = 0
Stenosis of the superior mesenteric artery	*n* = 1	*n* = 0

RHA = right hepatic artery, LHA = left hepatic artery.

## Data Availability

The datasets used and analyzed during the current study are available from the corresponding author upon reasonable request.
